# Characterization of Membrane-Associated Progesterone Receptor Component-2 *(MAPRC2)* from *Trichinella spiralis* and Its Interaction with Progesterone and Mifepristone

**DOI:** 10.3390/vaccines9080934

**Published:** 2021-08-23

**Authors:** Muhammad Tahir Aleem, Jiawen Shi, Zhengqing Yu, Zhaohai Wen, Yang Zhang, Meng Liang, Shakeel Ahmed Lakho, Muhammad Haseeb, Haider Ali, Muhammad Waqas Hassan, Xiaokai Song, Xiangrui Li, Lixin Xu, Ruofeng Yan

**Affiliations:** MOE Joint International Research Laboratory of Animal Health and Food Safety, College of Veterinary Medicine, Nanjing Agricultural University, Nanjing 210095, China; 2018207076@njau.edu.cn (M.T.A.); 2019107080@njau.edu.cn (J.S.); 2018207044@njau.edu.cn (Z.Y.); 2019207057@njau.edu.cn (Z.W.); 2018207018@njau.edu.cn (Y.Z.); 2018207024@njau.edu.cn (M.L.); 2017207046@njau.edu.cn (S.A.L.); 2016207041@njau.edu.cn (M.H.); 2018207074@njau.edu.cn (H.A.); 2015207037@njau.edu.cn (M.W.H.); songxiaokai@njau.edu.cn (X.S.); lixiangrui@njau.edu.cn (X.L.); xulixin@njau.edu.cn (L.X.)

**Keywords:** hormones, *MAPRC2*, mifepristone, progesterone, progesterone receptor, *Trichinella spiralis*

## Abstract

*Trichinellosis* is a foodborne zoonotic disease caused by *Trichinella* spp., including *Trichinella spiralis*. In the present study, *T. spiralis* membrane-associated progesterone receptor component-2 (*Ts-MAPRC2*) gene was cloned and characterized using protein sequencing analysis. Furthermore, the expression, purification, immunoblot assay, binding ability with progesterone antibody, and immunofluorescence assay were performed. A direct effect of progesterone (P4) and mifepristone (RU486) on the *Ts-MAPRC2* gene was determined using in vitro cell culture that showed different expression levels at all developmental stages (muscle larvae (ML), female adult worm (F-AL), male adult worm (M-AL), and newborn larvae (NBL)). Subsequently, the in vitro phenotypic effects of P4, RU486, and *rTs-MAPRC2-Ab* on F-AL and ML stages were measured. Later, the in vivo phenotypic effect and relative mRNA expression of mifepristone on the F-AL stage were studied. Our results revealed that the *Ts-MAPRC2* gene is critical to maintaining pregnancy in the female adult worm (F-AL) of *T. spiralis*. The 300 ng/mL of *P4* and 100 ng/mL of RU486 showed downregulation of the *Ts-MAPRC2* gene in F-AL (*p* ≤ 0.05). This plays an important role in abortion and possibly decreases the worm burden of *T. spiralis* in the host. Only 30 ng/mL *P4* showed significant upregulation in F-AL (*p* ≤ 0.05). The current study provides new insights regarding the antihormone (P4 and RU486) drug design and vaccine therapy of recombinant *(rTs-MAPRC2)* protein as well as their combined effects to control *T. spiralis* infection.

## 1. Introduction

*Trichinellosis* is an important foodborne zoonotic disease caused by a nematode parasite named *Trichinella spiralis* (*T. Spiralis*). It ranks seventh among the world’s most infectious parasitic diseases [1]. The main source of *T.* *spiralis* infection in humans is pork, in various forms (i.e., raw, undercooked, etc.), and its byproducts [2,3,4]. Since China is the leading consumer of pork and its byproducts in the world, their high morbidity from this disease due to the expanded dissemination of naturalized animal reserves is becoming an increasingly serious issue [5,6,7]. Although antihelminthic agents are extensively practiced against trichinellosis, their excessive use triggers the emergence of drug residues in meat, drug resistance in the parasite, and adverse effects the environment as well. Thus, the advancement of an efficient vaccine against *Trichinellosis*, especially for humans and pigs, is a potential measure to prevent infection [7,8]. Recently, a series of proteins participated against host irruption, the viability of parasite, and, therefore, produced resistance as vaccine applicants. Furthermore, their defensive effect that resists *T. spiralis* larvae to inoculate in model animals has been examined [9,10,11,12]. Many types of these vaccines showed relatively positive action against *T. spiralis* infection; however, no such vaccine that provides adequate immunity against *T. spiralis* infection is commercially available [1]. Progesterone (*P4*) is a gonadal hormone synthesized in the female ovary, male adrenal cortex, and testes; high levels of progesterone are found in females, especially in the follicular phase of the menstrual cycle as compared to males [13]. *P4* also has immunosuppressive effects and shifts in immune response from type 1 T helper (Th1) to type 2 T helper (Th2) cells [14,15]. Anzaldúa et al. [16] studied the highest level of *P4* that causes resistance against parasite invasion during pregnancy. Physiologically, the association between the increase in the *P4* doses and induction of NBL mortality was observed in *T. spiralis* and *Trichinella zimbabwensis* [17,18].

Mifepristone (*RU486*) works as an antagonist against the progesterone receptor (*PR*) and glucocorticoid receptor (*GR*) with abortifacient and anticancer activities [19]. In the case of helminths, several research studies are focused on *PGRMC* receptors [20]. Likewise, *RU486* was one of the first medications approved for surgical abortion; it is often used to terminate an early or midterm pregnancy [21]. Hence, *PR* and binding type of *P4* molecules (agonist) and *RU486* (antagonist) might be helpful to elaborate *T. spiralis* species regarding differentiation and reproductive development as well as creating potential pharmacological targets that might be used as a drug therapy against *Trichinellosis*.

The purpose of this study was to clone and characterize the *T. spiralis* membrane-associated progesterone receptor component-2 (*Ts-MAPRC2*) gene. Subsequently, we studied the protein sequencing, expression, purification, immunoblot assay, immunofluorescence assay (IFA), and binding ability with progesterone (*P4*). Furthermore, in this study, we used an in vitro cell culture technique to determine the direct effect of *P4* and *RU486* on *Ts-MAPRC2***,** which showed different expression levels of *Ts-MAPRC2* gene at all developmental stages (female adult worm (F-AL), male adult worm (M-AL), muscle larvae (ML), and newborn larvae (NBL)) of *T. spiralis*. Similarly, the in vitro phenotypic effect of *P4*, *RU486*, and *rTs-MAPRC2-Ab* (rat antisera against *rTs-MAPRC2*) on F-AL and ML stages were observed. Additionally, the in vivo phenotypic effect and relative mRNA expression of *RU486* on the F-AL stage were studied. This approach will help to design new antihormone (*P4* and *RU486*) drug and vaccine therapy of recombinant (*rTs-MAPRC2*) protein with their combined effect to control *Trichinellosis*.

## 2. Materials and Methods

### 2.1. Animals and Parasites Preservation

BALB/c mice (body weight 18–20 g) and SD (Sprague–Dawley) rats (body weight 220–250 g) were bought from the Qinglongshan animal breeding farm in Nanjing, Jiangsu, China (certified: SCXK 2008–0004). The animals were maintained under supervised conditions by the Animal House of Nanjing Agricultural University. *T. spiralis* (ISS534) was isolated from a pig in Nanyang, Henan Province, China, and preserved by serial passage in BALB/c mice after every 6–8 months. *T. spiralis* muscle larvae (ML) was recovered from BALB/c mice by 40 dpi (days post-infection) with a standardized HCl–pepsin digestion technique [22]. Adult worms (AL) were retrieved from the intestine at 6 dpi, and newborn larvae (NBL) were recovered from a female adult at 6 dpi from the RPMI-1640 culture media at 37 °C for 24 h as previously described by [23,24]. The parasites collected at different development phases were homogenized and chilled immediately in liquid nitrogen.

### 2.2. Bioinformatics Analysis and Molecular Modeling

The predicted whole-genome coding sequence of the *T. spiralis (Ts)* membrane-associated progesterone receptor component-2 *(Ts-MAPRC2)* gene (XM_003375886.1) was used in this study (http://www.ncbi.nlm.nih.gov, accessed on 3 January 2020). Expasy website (http://www.expasy.org, accessed on 10 January 2020) was used to study sequence properties of *Ts-MAPRC2*. The phylogenetic analysis of the *MAPRC2* (*T. spiralis*) protein sequence was carried out with homologs genes from the other strains using the Clustal Omega (https://www.ebi.ac.uk/Tools/msa/clustalo, accessed on 12 January 2020). Prediction of transmembrane in protein and N-terminal signal peptide prediction were confirmed by online tools TMHMM (http://www.cbs.dtu.dk/services/TMHMM, accessed on 14 January 2020) and SignalP-5.0 Server (http://www.cbs.dtu.dk/services/SignalP, accessed on 14 January 2020). Moreover, we predicted the three-dimensional pattern of the homodimer using homology modeling (https://swissmodel.expasy.org/interactive/HnydV2/templates, accessed on 16 August 2021).

### 2.3. Cloning of Ts-MAPRC2

The full-length sequence of *Ts-MAPRC2* (705 bp) comprises 234 amino acids. Fragment size of 97–234 aa (225–705bp) from the *Ts-MAPRC2* having conserved domain (104–173 aa) was expressed in the current study. The *Ts-MAPRC2* gene of *T. spiralis* was amplified using qRT-PCR analysis. Specific primer (5′-GAATTCAATAGATTTCGTATAAAATGGACATCT-3′) and (5′-AAGCTTTCACTGATCATCAACATCACAATCAGAG-3) with restriction enzymes *EcoR I* and *Hind III* was used to clone our gene. The PCR product was purified by Gel-Extraction Kit (Omega, Biotech, Norcross, GA, USA) and ligated into a pMD19-T cloning vector (TaKaRa, Dalian, China). The recombinant (pMD19-T/*Ts-MAPRC2*) plasmid was further processed into the *E. coli* (DH5α) strain (Invitrogen, Shanghai, China) and cultivated in LB (Luria Bertani) [25] medium with ampicillin (100 μg/mL). Positive bacterial (pMD19-T/ *Ts-MAPRC2*) clones were assured by digestion of restriction enzymes and confirmed through sequencing (Invitrogen, China).

### 2.4. Development of Recombinant Ts-MAPRC2 (rTs-MAPRC2)

The restriction digestion of the plasmid (pMD19-T/ *Ts-MAPRC2*) was carried out using the enzymes viz., *EcoR I* and *Hind III* was cloned into the prokaryotic expression vector pET-32a (+) (Novagen, Beijing, China). Recombinant plasmid (pET32a (+)/*Ts-MAPRC2*) was then processed into BL21 (DE3) and induced protein expression by 1 mM IPTG (Isopropyl-β-D-thiogalactopyranoside) (Sigma-Aldrich, Shanghai, China). Further, cells were harvested and lysed by lysozyme (10 μg/mL) (Sigma-Aldrich, Kenilworth, NJ, USA), and by sonication. The sonicated outputs were subsequently confirmed on the SDS-PAGE (12% *w/v*). Recombinant *Ts-MAPRC2* (*rTs-MAPRC2*) protein was purified by the His-TrapTM FF column following the manufacturer’s instructions (GE Healthcare, Piscataway, NJ, USA), and protein concentration was calculated by Pierce-TM BCA-Protein Assay Kit (Thermo Scientific, Waltham, MA, USA). Empty pET32a (histidine-tagged) protein was purified and expressed using the same procedure mentioned above for *Ts-MAPRC2* and utilized vector-protein as a negative control. Pictures of the SDS-PAGE carrying the r*Ts-MAPRC2* purified protein were taken. The stock of *rTs-MAPRC2* protein was prepared and preserved at −80 °C until the next experiments.

### 2.5. Generation of the Rat-Polyclonal Antibody of rTs-MAPRC2

For antisera preparation, SD rats (*n* = 6) were immunized subcutaneously with *rTs-MAPRC2* protein (300 μg) combined with Freund’s complete adjuvant (Sigma–Aldrich, Darmstadt, Germany) equally. After two weeks, the second dose of 200 μg *rTs-MAPRC2* protein was inoculated with Freund’s Incomplete Adjuvant (Sigma-Aldrich). Again, we repeated the same two booster doses at an interval of one week. After one week of the last dose, serum samples were collected and stored at −80 °C for further experiments. Serum collected from nontreated rats (*n* = 3) was used as a negative control.

### 2.6. Immuno-Blot Assay of rTs-MAPRC2

Separation of *rTs*-MAPRC2 protein carried by SDS-PAGE (12%), and were transferred to nitrocellulose membrane (Millipore, Bedford, MA, USA). Afterward, the membrane was blocked with skim milk (5% *w/v*) powder in TBST (0.5% Tween-20 in TBS) at 37 °C for 2 h. Then, washed three times with TBST, and membrane again incubated with primary antibody (rat antisera against *rTs*-MAPRC2) at 37 °C for 1 h (1:300 dilution). Normal rat serum was used as a negative control. Following this, the strips were washed three times and again incubated with secondary antibody-conjugated horseradish peroxidase (HRP) goat-antirat IgG (1:3000 dilutions) (Sigma, St. Louis, MO, USA) for 2 h at 37 °C. In the end, the immune-reaction were appeared within 3–5 min, as per the manufacturer’s directions of the DAB-HRP color development kit (Beyotime, Nantong, Jiangsu, China).

### 2.7. Identification of Native Ts-MAPRC2 Protein by Western Blot Assay

The total soluble protein of *T. spiralis* (muscle larvae) parasites was collected using RIPA solution (Thermo Scientific, Waltham, MA, USA) and a proteinase inhibitor (Thermo Scientific, Waltham, MA, USA) for complete lysis. The solution, containing the total soluble protein of *T. spiralis* was collected by high-speed centrifugation at 12,000 r/min for 30 min at 4 °C, and the supernatant was determined by the bicinchoninic acid method (BCA) kit mentioned above [25]. Afterwards, the total soluble proteins of *T. spiralis* (muscle larvae) parasites were carried by SDS-PAGE (12%) and transferred to polyvinylidene difluoride (PVDF) membranes (Millipore, Bedford, MA, USA) as described above. Then nonspecific binding was blocked with 5% skim milk in Tris-buffered saline containing 0.1% Tween-20 (TBST). The membranes were washed three times with TBST and incubated with primary antibody (rat antisera against *rTs*-MAPRC2,1:300 dilution) at 4 °C for overnight. Afterward, the membranes were washed three times and incubated with HRP-conjugated goat-antirat IgG (diluted 1:5000 in TBST) for 1 h at 37 °C. Finally, the Tanon™ High-sig ECL Western Blotting Substrate kit was used to detect bound antibodies according to the manufacturer’s instructions [26].

### 2.8. Determine the Binding Ability of rTs-MAPRC2 Protein with Progesterone

In order to determine the binding ability of *rTs-MAPRC2* protein with progesterone, we used the progesterone antibody (PROG-Ab) kit (Sandwich ELISA kit, FY95030-B, Feiya-Biotechnology, Nantong, Jiangsu, China). The pET-32a and PBS (phosphate buffer saline) were used as a control for the relative comparison. Initially, a standard curve was prepared using different concentrations (ng/mL) of the standard sample. According to kit instructions, 50 μL of the standard sample was added into three wells. Sample diluent (40 μL) was combined with *rTs-MAPRC2* protein (10 μL) and pET-32a (10 μL) protein and incubated at 37 °C for 30 min. After this, it was washed with the diluted washing solution (20×) 5 times for 30 s. Then, 50 Μl of HRP-Conjugate solution was poured into each well and incubated again at 37 °C for 30 min. Again, it was washed with the diluted washing solution (20×) 5 times for 30 s. Afterward, the 50 μL of Chromogen-A and Chromogen-B were added and incubated for 10 min at 37 °C. Finally, 50 μL of a stop solution was added, and the color change from blue to yellow was observed. The optimum density (OD) values were measured at 450 nm with a plate reader and compared (Thermo Fisher, Life Technologies, Waltham, MA, USA).

### 2.9. Immunofluorescent Assay of rTs-MAPRC2 at Different Developmental Stages

The cross-section of *T. spiralis* samples at different developmental stages, i.e., ML, F-AL, M-AL, and NBL were fixed in 4% formaldehyde-0.2% glutaraldehyde with PBS for 45 min, and snap frozen in liquid nitrogen. With the use of a cryotome (CM1950-Frankfurt, Germany), worms were cut into cross-section pieces (10 μm thick) and cleansed by PBS. First, the 5% BSA (Bovine serum albumin) was treated with slides to block nonspecific binding and continued by incubation with primary antibody (rat antisera against *rTs-MAPRC2*) as well as normal rat serum (control group) at 37 °C for 2 h (1:300 dilution). Then, both group slides were washed with PBS and incubated with Cy3-goat labeled antirat as a secondary antibody (Beyotime-Shanghai, China) for 1h at 37 °C. Thereafter, the DAPI (diamidino-2-phenylindole) (Sigma, USA) stain was used for staining the nuclei of the worm cells for 5 min under darkness. Lastly, a laser confocal microscope (PerkinElmer, Boston, MA, USA) was used to observed worm cross-section cells.

### 2.10. Relative mRNA Expression of Ts-MAPRC2 Gene at Different Stages Incubated with Progesterone (P4) and Mifepristone (RU486)

The powder form of progesterone (*P4*) and mifepristone (*RU486*) were purchased from Sigma (Sigma-Aldrich, St. Louis, MO, USA), and dissolved in absolute ethanol to prepare the desired stock solution following company instructions. Stock solutions were sterilized using the filtration process with a 0.2 mm Millipore filter. The highest level of progesterone was 100–200 ng/mL in rats, 81.9 ng/mL in mice, 25–30 ng/mL in pigs, and 25 ng/mL in pregnant women and were determined [27,28,29,30]. So, five different concentration of agonist progesterone (P300 ng/mL, P100 ng/mL, P30 ng/mL, P10 ng/mL, and P3 ng/mL) and also three concentrations of antagonist mifepristone (M300 ng/mL, M100 ng/mL, and M30 ng/mL) were prepared. Control (only RPMI), and control vehicle ethanol (EtOH-RPMI) (0.125%) were prepared as described by [31]. All stages of the parasites viz., F-AL, M-AL, ML, and NBL were collected as described previously by [23,24]. The 2000 worms/well at all above parasite stages (ML, AL, NBL) were cultured in 6-well plates, and a 6-well precondition was used. The cultured medium consisted of RPMI-1640, 10% heat-inactivated FBS (Fetal bovine serum), and 2% antibiotics (100 U/mL penicillin; 100 mg/mL streptomycin) (Gibco, Paisley, UK). The medium was incubated at 37 °C and 5% of CO_2_ for 48 h and changed after 24 h for all treatments. The female (F-AL) and male (M-AL) adult worms were separated under light microscopy using Axiovert Zeiss Microscope (25× Neo Plan objectives). The expression of the *Ts-MAPRC2* gene in all treated groups at various developmental stages was measured by relative quantitative PCR (RT-qPCR) as previously described by [31]. Briefly, the Trizol technique was used for RNA extraction from treated groups at all stages of worms using a prime script RT reagent kit (Takara, Berkeley, CA, USA). The isolated RNA from each group at all parasite stages was reverse transcribed using HiScript II Q RT SuperMix (Vazyme, Nanjing, China) kit using manufacturer instructions. The *Ts-MAPRC2* gene-specific primer was used as follows: forward (5′-ACGATGTGACCCGAAAGAGA-3′) and reverse (5′-CATGCATAGCCCATTCACGT-3′). Quantitative amplifications were performed by BI 7500 Fast Real-time PCR System (Applied Biosystem, Waltham, MA, USA) with the use of Cham-QTM SYBR RT-qPCR master mix-Kit (Vazyme, Nanjing, China). GenBank Accession No. AF452239 *GADPH* (Glyceraldehyde-3-phosphate dehydrogenase) of *Trichinella* was used as an internal control. The *GADPH* primer was designed as following; forward (5′-GTCGTGGCTGTGAATGATC-3′) and reverse (5′-GCTGCCCCACTTAATTGCTT-3′), and data were computed using the comparative Ct (2^−ΔΔCt^) technique [32].

### 2.11. In Vitro Phenotypic Effect of P4, RU486, and rTs-MAPRC2-Ab on F-AL and ML Stages

The selected concentration of progesterone (P30 ng/mL), mifepristone (M100 ng/mL), and *rTs-MAPRC2-Ab* (rat antisera against *rTs-MAPRC2*) ratio (1:300 dilution) were used at F-AL and ML stages with both controls (only RPMI and EtOH-RPMI), collected as described above, and the previously described procedures were followed according to [23,24]. The 2000 worms/well of both stages (F-AL, ML) were cultured in a 6-well plate, and a 6-well precondition was used. The cultured medium consists of RPMI-1640, 10% heat-inactivated FBS (Fetal bovine serum), and 2% antibiotics (100 U/mL penicillin; 100 mg/mL streptomycin) (Gibco, Paisley, UK), and incubated at 37 °C and 5% CO_2_ for 48 h and the medium changed after 24 h for all treatments. The female (F-AL) and male (M-AL) adult worms were separated under light microscopy using Axiovert Zeiss Microscope (25× Neo Plan objectives) and observed the phenotypic appearance at both stages (F-AL and ML) using an inverted bright field microscope (Olympus, Shibuya, Japan). In the F-AL stage, the pregnancy maintenance or aborted to NBL were observed. While at the ML stage ecdysis (molting process) and motility of parasites were noted.

### 2.12. The Phenotypic Effect and Relative mRNA Expression of Mifepristone on F-AL Stage by In Vivo

To determine the phenotypic effect of mifepristone on F-AL (female adult worm) of *T. spiralis*, 30 BALB/c mice were divided into three groups (n = 10) (i) mifepristone (M100 ng/mL) administration, (ii) adjuvant group EtOH-IN (Olive oil-ethanol 3:1), and (iii) control group. Mifepristone (M100 ng/mL) powder from Sigma (Sigma-Aldrich, USA) was dissolved in a mixture of EtOH-IN and injected subcutaneously (SC) into BALB/c mice every 24 h till retrieved AL (Adult worms) from the intestine at 6 dpi (days post-infection) with 0.1 mL of the suspension comprising boost dose of steroid according to the protocol of González et al. [28]. In the adjuvant group EtOH-IN, BALB/c mice were administrated only adjuvant EtOH with the same protocol, and the control group was kept without any drug therapy. On day six of mifepristone (M100 ng/mL) treatment, all groups of mice have orally injected 500 *T. spiralis* (ML) with the help of gastric cannula [22]. Adult worms (AL) were collected from the intestine at 6 days post-infection (dpi) and adult worms (F-AL, M-AL) were separated using Axiovert Zeiss Microscope (25x Neo Plan objectives) and observed the phenotypic appearance, especially pregnancy, occurred or not using an inverted bright field microscope (Olympus, Shibuya, Japan). The gene (*Ts-MAPRC2*) expression of all three groups at the F-AL developmental stage was also measured by relative quantitative PCR (RT-qPCR) as described by [32].

### 2.13. Statistical Analysis

The data were analyzed by one-way analysis of variance (ANOVA) followed by Tukey’s and LSD (Least significant difference) using analytical statistics software Statistix 8.1. (2003) (Tallahassee, FL, USA). The RT-qPCR data were normalized using 2^−ΔΔCt^ in Microsoft Excel 2010 (Redmond, Washington, USA) and a T-test was used to compare between treatments (each sample replicated three times). Origin software (Origin Pro 2021, Origin Lab Corporation, Northampton, MA, USA) was used to perform figures. Statistical data were presented as mean ± SD (*n* = 3). *p* ≤ 0.05 was considered significant.

## 3. Results

### 3.1. Sequence Analysis of Ts-MAPRC2

**Figure 1:**
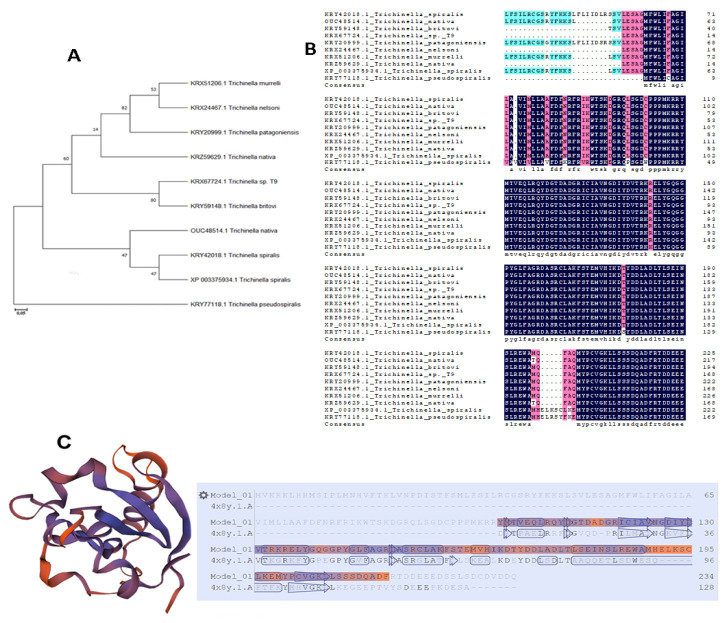
Phylogenetic tree and multiple sequence alignment. (**A**) A phylogenetic tree of *Ts-MAPRC2* and homologs with other species. (**B**) Multiple Sequence Alignment of *Ts-MAPRC2* by Clustal Omega with other *Trichinella* species. Different colors show the amino acid identity, and gaps represent the sequence with other *Trichinella* species, respectively. (**C**) The predicted *Ts-MAPRC2* 3D model from the crystal structure of the human PGRMC1 cytochrome b5-like domain (4 × 8y.1.A) with 42.50% of identity (Figure 1C).

The full-length gene sequence (Gene Bank Accession No. XM_003375886.1) of 705 bp was predicted to encode protein consisting of 234aa, as well as possess the cytochrome b5-like haem/steroid binding conserved domain of 104–173aa. Fragment size of 480 bp (between 225–705 bp) of the *Ts-*MAPRC2 gene were used for cloning that encodes the protein of 137aa (97–234aa) possessing the conserved domain of 6–75aa submitted online in NCBI (http://www.ncbi.nlm.nih.gov, accessed on 21 February 2020) and issued (22 December 2020) a specific accession number (MT093680). Bioinformatics online tools TMHMM revealed that there were no transmembrane and Signal peptides found in *Ts-MAPRC2* (MT093680). Percent matrix index showed more similarity of the *Ts-MAPRC2* with other *Trichinella* species strains. Phylogenetic tree and multiple sequence alignment of *Ts-MAPRC2* using the Clustal Omega with other *Trichinella* species are presented in Figure 1A,B. The best template was found by the Swiss model to predict the *Ts-MAPRC2* 3D model from the crystal structure of the human PGRMC1 cytochrome b5-like domain (4 × 8y.1.A) with 42.50% of identity (Figure 1C).

### 3.2. Cloning, Expression, Purification, Immunoblot of rTs-MAPRC2 and Native Ts-MAPRC2 Protein

**Figure 2:**
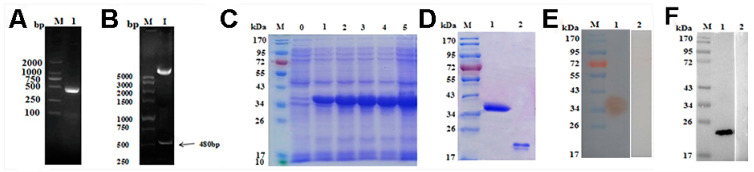
Cloning of Ts-MAPRC2 gene and identification of recombinant plasmid (pET-32a (+) Ts-MAPRC2. Lane M represents DNA marker (**A**) Lane 1; Ts-MAPRC2 gene amplified by PCR (**B**) Lane 1; Digested recombinant plasmid {(pET-32a (+)} Ts-MAPRC2 with EcoR1 and Hind III. Expression and purification of r*Ts-MAPRC2*. Lane M: Standard molecular weight of protein marker Lane 0: Recombinant expression vector prior IPTG (isopropyl-ß-d-thiogalactopyranoside) induction. (**C**) Lane 1-5: Expression of *rTs-MAPRC2* protein after IPTG induction at different time points. (**D**) Lane 1: Purified expression of the *rTs-MAPRC2* protein. Lane 2: Expression of pET-32a protein resolved on SDS-PAGE. (**E**) M: Standard marker molecular weight. Lane 1: Purified *rTs-MAPRC2* protein was transferred to the membrane and probed with SD rat serum immunized by *rTs-MAPRC2* protein. Lane 2: Membrane probed by normal rat serum as control. (**F**) Western blot analysis of native *Ts-MAPRC2* protein. Lane 1: *Ts-MAPRC2* native protein detected by anti-*rTs-MAPRC2* rat sera; Lane 2: no protein was detected with normal rat sera.

The amplified PCR product of the *Ts-MAPRC2* gene (480 bp) was procured successfully from cDNA of *T. spiralis* (Figure 2A) using the pair of primers and cloned into pMD-19T (cloned vector). Then transformed into pET-32a (expression vector) (Figure 2B) and confirmed by restriction enzymes digestion using the *EcoR I* and *Hind III.* Cloned *Ts-MAPRC2* gene (480bp) sequences were translated into 137aa, and molecular weight was estimated to 17.6 ≈ 18 kDa. The *rTs-MAPRC2* protein was expressed on SDS-PAGE (12%) with IPTG and observed high expression after five induction hours (Figure 2C). After purification, the molecular weight was showed around 38kDa (Figure 2D, Lane 1) along with 20 kDa mass of (poly his-tag pET-32a) expression vector (Figure 2D, Lane 2). Immuno-blot assay revealed that *rTs-MAPRC2* could be recognized by polyclonal antibodies produced in rat serum against the *rTs-MAPRC2* protein (Figure 2E, Lane 1), and compared with normal rat serum (Figure 2E, Lane 2). The native *Ts-MAPRC2* protein was recognized by anti-*rTs-MAPRC2* polyclonal antibodies (Figure 2F, Lane 1) and compared with normal rat serum (Figure 2F, Lane 2). However, the molecular weight of native *Ts-MAPRC2* protein was showed around 23.5 ≈ 24 kDa because the size of *Ts-MAPRC2* gene was 705 bp. The above result showed that *rTs-MAPRC2* as well as native *Ts-MAPRC2* protein possesses antigenicity, and easily recognized by the host immune system.

### 3.3. Binding Ability of rTs-MAPRC2 Protein by Sandwich ELISA

**Figure 3:**
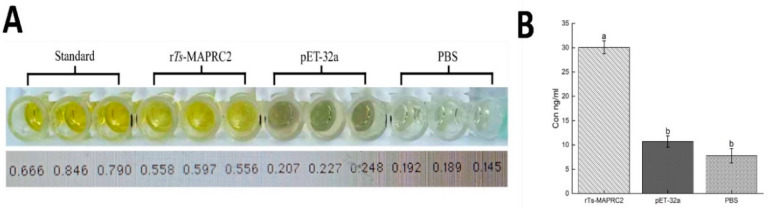
(**A**) The binding ability of *rTs-MAPRC2* protein compared to pET-32a and PBS as control using a (PROG-Ab) Sandwich ELISA kit (OD450). The data were shown in three independent trials. (**B**) Statistical comparison between the *rTs-MAPRC2* protein as treatment and control were performed using a Tuckey Test Significant differences were determined to a threshold of *p* < 0.05.

The binding capacity of *rTs-MAPRC2* protein with progesterone was compared with pET-32a and PBS as control. Our results revealed that there are highly significant differences (*p < 0.05*) between *rTs-MAPRC2* protein and both controls (pET-32a and PBS) (Figure 3) compared to the standard. But no significant difference was observed between pET-32a and PBS.

### 3.4. Immunofluorescent Assay (IFA) of Ts-MAPRC2 Gene at Various Developmental Stages

**Figure 4:**
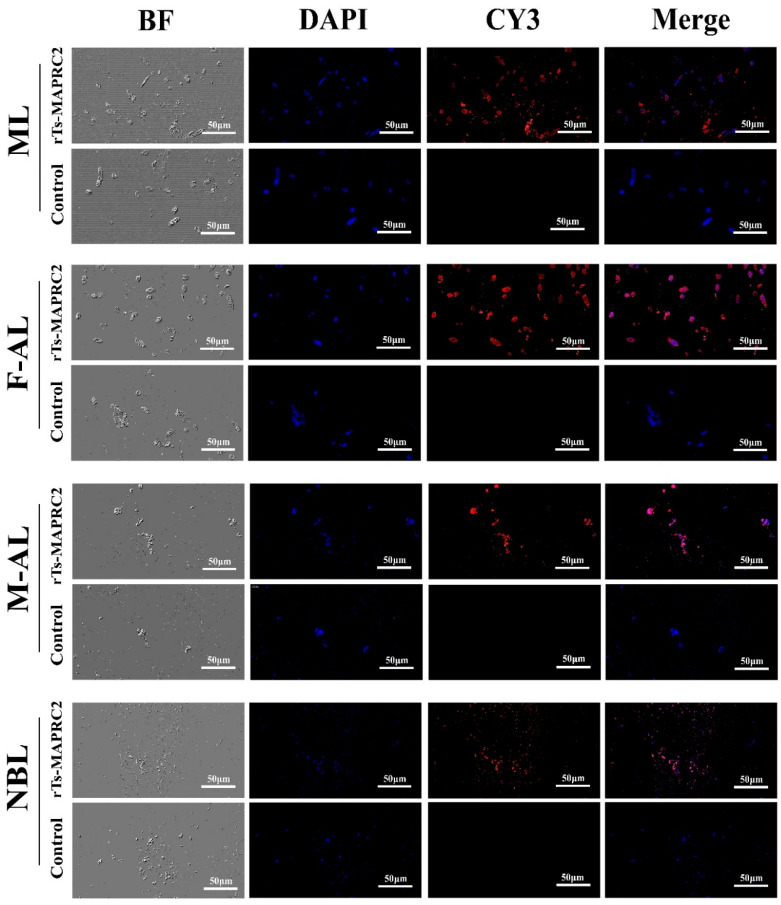
Immunolocalization of *Ts-MAPRC2* at different developmental stages of *T. spiralis*. Cross-section of Intact worms of all stages (F-AL, M-AL, ML, and NBL) were studied using IFA with anti-*rTs-MAPR* sera followed by BF (Bright field), DAPI, staining with Cy3-conjugated secondary antibody, and Merge. ML and F-AL stages showed high *Ts-MAPR* gene localization as compared to M-AL and NBL stages. Moreover, F-AL showed high immunolocalization compared to M-AL. No fluorescence was observed in control of all the worm’s developmental stages with a scale bar of 50 µm.

IFA was performed to determine the presence of the *Ts-MAPRC2* gene at different developmental stages using anti-*rTs-MAPRC2* rat serum, Cy3-conjugated secondary antibody, and DAPI (nuclei dye). The cross-section cells of all developmental stages **[**ML, F-AL, M-AL, and NBL**]** of *T. spiralis* were studied. As shown in Figure 4, Cy3-labeled *Ts-MAPRC2* gene, DAPI-labeled nuclei displayed red and blue fluorescence for all stages cross-section cells (ML, F-AL, M-AL, and NBL) respectively. ML and F-AL stages showed high *Ts-MAPRC2* gene localization as compared to M-AL and NBL stages. Moreover, F-AL showed high immunolocalization compared to M-AL. However, no red fluorescence was observed in all stages (ML, F-AL, M-AL, and NBL) of the control groups.

### 3.5. Relative mRNA Expression of Ts-MAPRC2 at all Stages by In Vitro Treatment of P4 and RU486

The transcriptional pattern of the *Ts-MAPRC2* gene in the parasite treated at different concentrations of P4 (P300 ng/mL, P100 ng/mL, P30 ng/mL, P10 ng/mL, and P3 ng/mL) as well as mifepristone (M300 ng/mL, M100 ng/mL, and M30 ng/mL) and their multi comparison analyses was investigated to find out the up-and down-regulation at different stages (F-AL, M-AL, ML, and NBL) using qRT-PCR with the transcription of GAPDH gene as a control.

#### 3.5.1. Comparison Between the Same Stage and Same Concentration of P4

**Figure 5:**
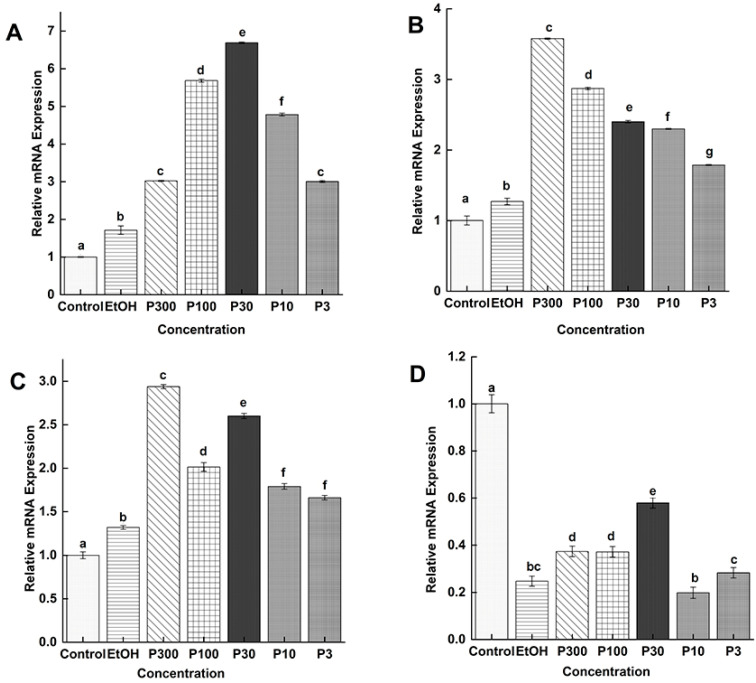
Relative mRNA levels of *Ts-MAPRC2* at various developmental phases of *T. spiralis*. The Comparison between the different concentrations of P4 with control (only RPMI) and control vehicle ethanol (EtOH-RPMI) among the same developmental stages (**A)** F-AL (**B**) M-AL (**C**) ML (**D**) NBL of *T. spiralis*. Statistical data were presented as mean ± SD. *p* ≤ 0.05, *p* ≤ 0.01 were considered significant. The same letters mean nonsignificant and the different letters mean significant.

The results of the comparative expression of the *Ts-MAPRC2* gene in the F-AL stage at different concentrations of P4 (P300 ng/mL, P100 ng/mL, P30 ng/mL, P10 ng/mL, and P3 ng/mL) with control (only RPMI) and control vehicle ethanol (EtOH-RPMI) are presented in Figure 5A. All concentrations of P4 (P300 ng/mL, P100 ng/mL, P30 ng/mL, P10 ng/mL, and P3 ng/mL) showed up-regulation of the *Ts-*MAPRC2 gene as compared to control (only RPMI) and control vehicle ethanol (EtOH-RPMI); in contrast, the P30 showed high up-regulation expression of the *Ts-MAPRC2* gene compared to all other concentrations with their both controls (only RPMI and EtOH-RPMI) (*p* ≤ 0.05). Figure 5B showed the comparative expression of *Ts-MAPRC2* gene in the M-AL stage of different concentrations of P4 (P300 ng/mL, P100 ng/mL, P30 ng/mL, and P10 ng/mL, P3 ng/mL) with control (only RPMI) and control vehicle ethanol (EtOH-RPMI). All concentrations of P4 showed up-regulation of the *Ts-*MAPRC2 gene relative to control (only RPMI) and control vehicle ethanol (EtOH-RPMI), but expression decreased in descending order from high concentration (P300 ng/mL) to low concentration (P3 ng/mL) compared with both controls (only RPMI and EtOH-RPMI) (*p* ≤ 0.05). Comparative expression of the *Ts-MAPRC2* gene in the ML stage at different concentrations of P4 with control (only RPMI) and control vehicle ethanol (EtOH-RPMI) are presented in Figure 5C. In the ML stage, all treatment concentrations showed up-regulation of the *Ts-*MAPRC2 gene, but P300 and P30 showed more upregulation compared with both controls (only RPMI and EtOH-RPMI) (*p* ≤ 0.01). Figure 5D shows the relative expression of the *Ts-MAPRC2* gene in the NBL stage at different concentrations of P4 with control (only RPMI) and control vehicle ethanol (EtOH-RPMI). All concentrations of P4 showed down-regulation compared with control (only RPMI), but here control vehicle ethanol (EtOH-RPMI) also showed down-regulation.

#### 3.5.2. Comparison Among the Same Stage and Same Concentration of Mifepristone (RU486)

**Figure 6:**
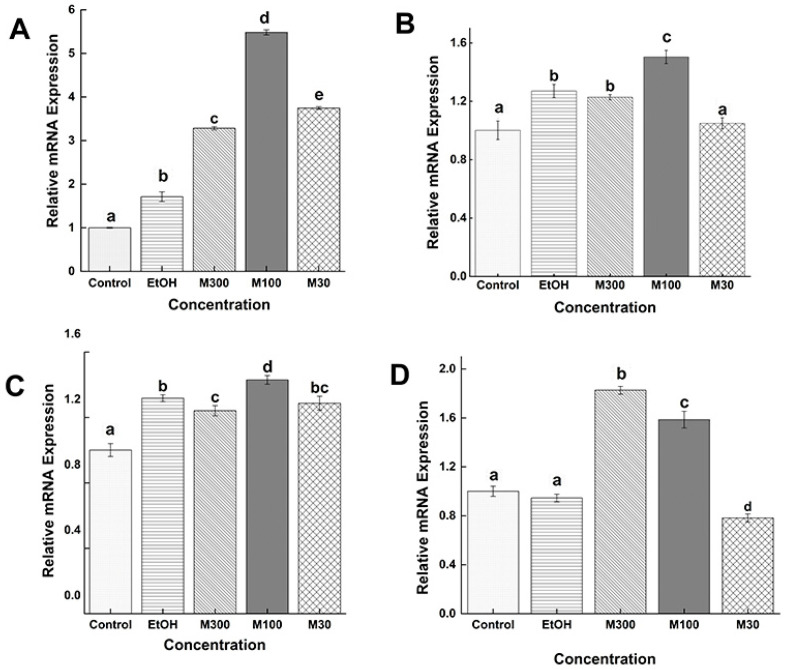
Relative mRNA levels of *Ts-MAPRC2* at various developmental phases of *T. spiralis*. The comparison between the different concentrations of mifepristone with **control** (only RPMI) and control vehicle ethanol (EtOH-RPMI) among the same developmental stages (**A**) F-AL (**B**) M-AL (**C**) ML (**D**) NBL of *T. spiralis*. Statistical data were presented as mean ± SD. *p* ≤ 0.05 was considered significant. The same letters mean nonsignificant and the different letters mean significant.

Relative expression of the *Ts-*MAPRC2 gene in the F-AL stage at different concentrations of mifepristone (M300 ng/mL, M100 ng/mL, and M30 ng/mL) with the control group (only RPMI) and control vehicle ethanol (EtOH-RPMI) are presented in Figure 6A. All concentrations showed up-regulation of the *Ts-*MAPRC2 gene as compared with control (only RPMI) and control vehicle ethanol (EtOH-RPMI), but M100 (ng/mL) showed high up-regulation expression of the *Ts-MAPRC2* gene compared to all other treatment concentrations with their both controls (only RPMI and EtOH-RPMI) (*p* ≤ 0.05). Figure 6B shows the comparative expression of the *Ts-MAPRC2* gene in the M-AL stage at different concentrations of mifepristone with control (only RPMI) and control vehicle ethanol (EtOH-RPMI). Here only M100 (ng/mL) showed up-regulation of the *Ts-*MAPRC2 gene as compared with control and control vehicle ethanol (EtOH-RPMI); however, the M300 (ng/mL) showed expression same as control vehicle ethanol (EtOH-RPMI), and the M30 (ng/mL) showed expression same as control (only RPMI) (*p* ≤ 0.05). Figure 6C shows the comparative expression of the *Ts-MAPRC2* gene in the ML stage at different concentrations of mifepristone with control (only RPMI) and control vehicle ethanol (EtOH-RPMI). In the ML stage, M100 (ng/mL) showed up-regulation of the *Ts-*MAPRC2 gene as compared with control (only RPMI) and control vehicle ethanol (EtOH-RPMI) but M300 (ng/mL) and M30 (ng/mL) showed almost the same expression compared with both controls (only RPMI and EtOH-RPMI) (*p* ≤ 0.05). Figure 6D shows the relative expression of the *Ts-MAPRC2* gene in the NBL stage at different concentrations of mifepristone (M300 ng/mL, M100 ng/mL, and M30 ng/mL) with control (only RPMI) and control vehicle ethanol (EtOH-RPMI). In the NBL stage, the expression decreased in descending order from high concentration (M300 ng/mL) to low concentration (M100 ng/mL) compared with both controls (only RPMI and EtOH-RPMI) (*p* ≤ 0.05). But (M30 ng/mL) showed downregulation respectively (*p* ≤ 0.05).

#### 3.5.3. Comparison Among the P4 and RU486 at F-AL Stage Using P30 and M30 as Controls

**Figure 7:**
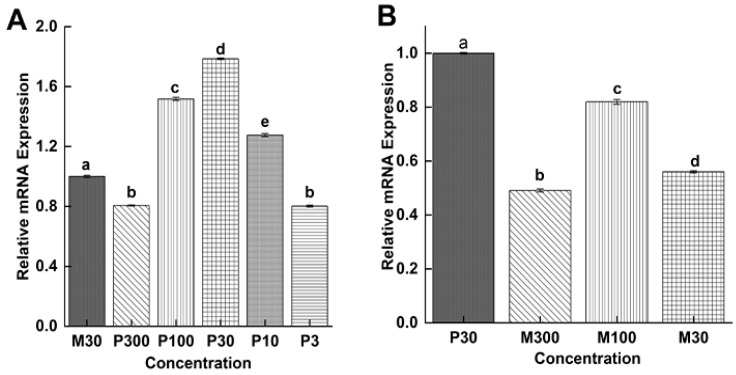
Relative mRNA expression of ***Ts-MAPRC2*** at F-AL (Female adult worm) phase of *T. spiralis*. (**A**) Comparison between the different concentrations of P4 with M30 ng/mL as control. (**B**) Comparison between the different concentrations of mifepristone with P30 ng/mL as control. Statistical data were presented as mean ± SD. *p* ≤ 0.05 was considered significant. The same letter means nonsignificant and different letters mean significant.

Relative expression analysis of the *Ts-*MAPRC2 gene in the F-AL stage at different concentrations of P4 with M30 ng/mL as control is presented in Figure 7A. P100 ng/mL, P30 ng/mL, and P10 ng/mL showed up-regulation of the *Ts-*MAPRC2 gene while P300 ng/mL, and P3 ng/mL showed down-regulation as compared with M30 ng/mL as control. Figure 7B shows the comparison expression of the *Ts-*MAPRC2 gene in the F-AL (Female adult worm) stage at different concentrations of mifepristone with P30 ng/mL as control. Here, all concentrations of mifepristone showed down-regulation compared with P30 ng/mL as control (*p* ≤ 0.05).

### 3.6. In Vitro Phenotypic Effect of P4, RU486, and rTs-MAPRC2-Ab on F-AL and ML Stages

**Figure 8:**
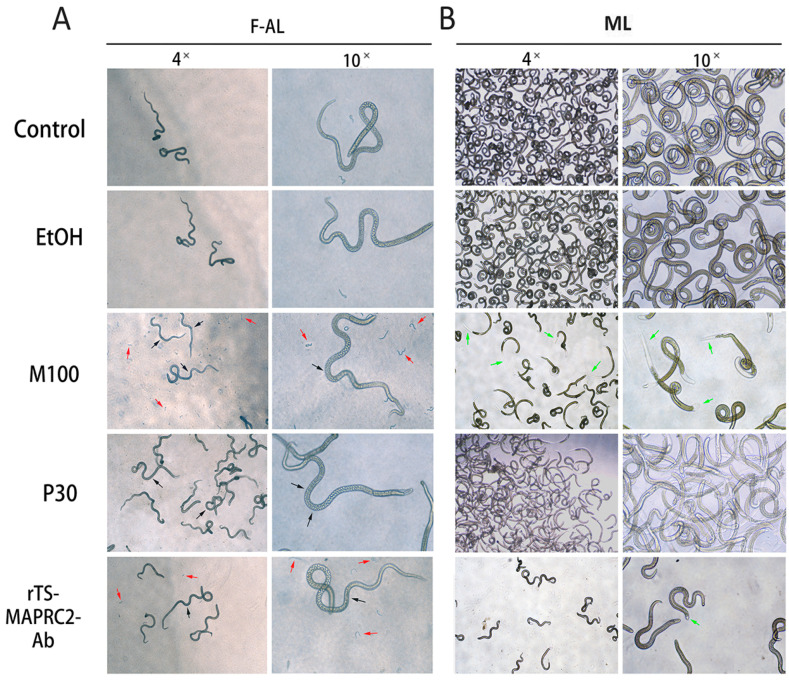
The in vitro phenotypic effect of P30 (ng/mL), M100(ng/mL), and r*Ts-MAPRC2*-Ab on F-AL (female adult worm) and ML (muscle larvae) stages were observed. (**A**) Compared the phenotypic appearance of pregnant female adult worm (F-AL) among P30 (ng/mL), M100(ng/mL), and r*Ts-MAPRC2*-Ab with both controls (only RPMI and EtOH-RPMI). Black arrows presented a female pregnancy site while red arrows present newborn larvae (NBL) at objectives 4× and 10×. (**B**) Compared the phenotype appearance of muscle larvae (ML) among P30 (ng/mL), M100(ng/mL), and r*Ts-MAPRC2*-Ab with both controls (only RPMI and EtOH-RPMI) to observed ecdysis (molting process is shown by green arrow) and motility at objective 4× and 10×.

In vitro phenotypic effect of P30 ng/mL, M100 ng/mL, and *rTs-MAPRC2-Ab* on the F-AL stage revealed that observed pregnancy and abortion to produce NBL parasites (Figure 8A). M100 (ng/mL) and *rTs-MAPRC2-Ab* showed more aborted NBL parasites as compared to controls (only RPMI and EtOH-RPMI), while P30 ng/mL prolonged pregnancy period and delays abortion to NBL compared with controls (only RPMI and EtOH-RPMI). Figure 8B shows the in vitro phenotypic effect of P30 ng/mL, M100 ng/mL, and *rTs-MAPRC2-Ab* on the ML stage. Here, the M100 has more ecdysis (molting process) and motility compared with P30 (ng/mL) concentration, *rTs-MAPRC2-Ab*, and controls (only RPMI and EtOH-RPMI). While *rTs-MAPRC2-Ab* also has high motility compared to P30 ng/mL, and both controls (only RPMI and EtOH-RPMI).

### 3.7. Phenotypic Effect and Relative mRNA Expression of Mifepristone on F-AL Stage by In Vivo

**Figure 9:**
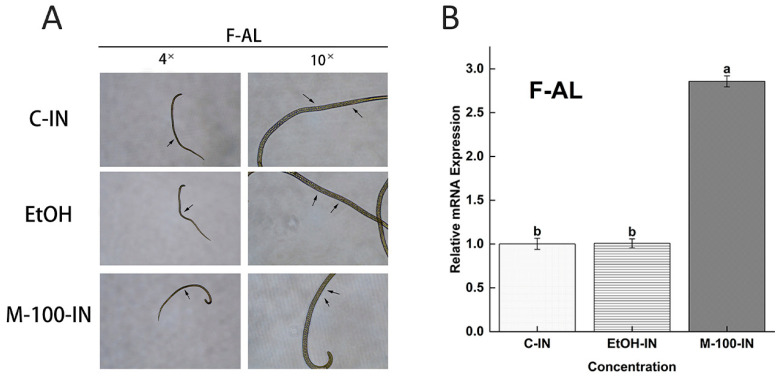
The in vivo phenotypic effect and relative mRNA expression of mifepristone (M100 ng/mL) on F-AL (female adult worm) were observed. (**A**) Compared the phenotypic appearance of early pregnancy stage of female adult worm (F-AL) among M100(ng/mL) with control and adjuvants EtOH-IN (olive oil-ethanol) at objective 4× and 10×. (**B**) Relative mRNA expression of *Ts-MAPRC2* at F-AL (female adult worm) phase of *T. spiralis* by in vivo study. Here, a comparison between M100 (ng/mL) with a simple control group and adjuvants EtOH-IN (Olive oil-ethanol) group. Statistical data were presented as mean ± SD. *p* ≤ 0.05 was considered significant. The same letters mean nonsignificant and different letters mean significant.

In vivo phenotypic effect of mifepristone (M100 ng/mL) on F-AL (female adult worm) are presented in Figure 9A. Comparison of the early pregnancy stage of F-AL among M100 ng/mL, EtOH-IN (Olive oil-ethanol) with the simple control mice groups. The simple control group and EtOH-IN showed normal pregnancy at 6 dpi (days post-infection), but the F-AL of the M100 ng/mL treated mice group did not show pregnancy and only see embryos inside of the F-AL body. In vivo study shows the relative mRNA expression of *Ts-MAPRC2* at F-AL phase of *T. spiralis* among three groups such as M100 ng/mL, EtOH (Olive oil-ethanol) with the simple control Figure 9B. M100 ng/mL showed up-regulation of the *Ts-*MAPRC2 gene as compared with EtOH-IN with the simple control (*p* ≤ 0.05).

## 4. Discussion

Progesterone receptor membrane components 1 and 2 *(PGRMC-1* and *PGRMC-2)* belong to the same family of MAPR (membrane-associated progesterone receptor) proteins [33,34], and *PGRMC-1* protein was collected first from smooth muscle of porcine with 28 kDa [35,36]. Likewise, in helminths, the authors of several studies reported PGRMC receptors, progestin-induced proteins, p48 progesterone-receptor-associated proteins, and small androgen receptor-interacting proteins that were found in *S. japonicum* [37,38].

In this study, we cloned, expressed, and evaluated the *MAPRC2* gene in *T. spiralis*. Furthermore, its expression levels with P4 (agonist) and RU486 (antagonist) hormones were also determined. Cytochrome-b5 haem/steroid binding domain present inside the *Ts-MAPRC2* protein (6–75aa) is calculated with BLAST and predicted 3D model from the crystal structure of the human PGRMC1 cytochrome b5-like domain with 42.50% of identity by Swiss model (Figure 1C). An online bioinformatics tool (TMHMM) shows that there was not any transmembrane region found in the cloned sequence (225–705bp). Notably, the percent matrix index displayed more resemblance between the *Ts-MAPRC2* and strains of other *Trichinella* species (Figure 1A,B). Interestingly, we magnificently cloned the *Ts-MAPRC2* gene, and its protein expression was confirmed via SDS-PAGE. The host immune system recognized both *rTs-MAPRC2* protein as well as native *Ts-MAPRC2* protein, confirmed by immunoblot assay suggested that the recombinant protein as well as native *Ts-MAPRC2* protein has the compatible antigenic features (Figure 2). Moreover, a binding capacity of *rTs-MAPRC2* protein with a progesterone antibody (PROG-Ab) was determined by Sandwich ELISA kit (Figure 3). This finding complements the idea that *rTs-MAPRC2* protein has a specific steroid-binding domain that might arbitrate the P4 effect in the reproductive process of *T. spiralis*. Similarly, *PGRMC-2* was detected mainly in reproductive parts of mammals (ovary, endometrium, placenta) associated with multiple cellular functions (differentiation, proliferation, and maturation), and also identified in *T. solium* cysticerci [33,39,40,41].

Furthermore, we examined the localization of the *MAPRC2* gene in *T. spiralis* by immunofluorescence staining at all developmental stages which confirmed the presence of *MAPRC2* gene throughout all the developmental stages (Figure 4). Other studies also performed the immunofluorescence assay to examine membrane-binding progesterone protein in the *T. solium* cysticerci [20].

The in vitro study shows relative mRNA expression of *Ts-MAPRC2* gene at all stages treated with different concentrations of progesterone, mifepristone, and their controls (ETH-RPMI and only RPMI) (*p* ≤ 0.05). Figure 5 shows the comparative expression of the *Ts-*MAPRC2 gene in all stages at different concentrations of P4 with a control group (*p* ≤ 0.05). All concentrations of P4 showed up-regulation of the target gene in three stages (F-AL, M-AL, and ML) among which F-Al P30ng/mL showed the highest expression level, but NBL showed down-regulation as compared to the control group (*p* ≤ 0.05). From these results, we can purpose that increase in the concentration of P4 from 300ng/mL results in downregulation; this strongly emphasizes the maximum levels of progesterone range 100–200 ng/mL in rats affect the death of newborn larvae in *T. spiralis* [17].

Figure 6 shows the comparative expression of the *Ts-MAPRC2* gene in all stages at different doses of mifepristone with controls. In the F-AL stage, only M100 ng/mL showed high up-regulation expression of the *Ts-MAPRC2* gene that confirms this concentration is good as antagonists against progesterone receptor (PR) as well as abortifacient activities [19]. Further, we confirmed through a cross-comparison expression of the *Ts-*MAPRC2 gene among the P4 and RU486 at the F-AL stage by P30 ng/mL and M30ng/mL used as controls. Figure 7A shows that P100 ng/mL, P30 ng/mL, and P10 ng/mL result in upregulation, while P300 ng/mL, P3ng/mL showed downregulation with M30 ng/mL as control, but it was noted that P30 ng/mL still shows high up-regulation. Figure 7B shows that all concentrations of mifepristone caused down-regulation compared to P30 ng/mL as control (*p* ≤ 0.05). Finally, we concluded that the progesterone (P4) upregulates the *Ts-MAPRC2* gene at a low concentration of P30 ng/mL but downregulate at P300 ng/mL, in the F-AL stage compared with M30 ng/mL positive control, this corroborated with the findings of Nuñez et al. [17] indicating more resistance against *T. spiralis* due to a high progesterone level compared with virgin rats. In vitro trials showed that pregnant rat sera could interrupt mortality of NBL of *T. spiralis* [17]. Although, mifepristone (antagonist) downregulated the *Ts-MAPRC2* gene at all concentrations compared with P30 ng/mL as a positive control which claims that the mifepristone (RU486) functions as antagonists against progesterone receptor (PR) and abortifacient activities [19].

Based on this analysis, we subsequently examined the phenotypic appearance of pregnant F-AL among P30 (ng/mL), M100(ng/mL), and *rTs-MAPRC2-Ab* with controls by in vitro. M100 ng/mL and *rTs-MAPRC2-Ab* showed more aborted NBL parasites as compared to control, while P30 ng/mL prolongs pregnancy and delays in abortion to produce NBL compared with control groups (see Figure 8A). These findings are in line with the findings of Nuñez et al. and Chen et al. [17,19]. Figure 8B shows that M100 ng/mL has more ecdysis (molting process) and motility compared with P30 ng/mL concentration, *rTs-MAPRC2-Ab* as compared to control. While *rTs-MAPRC2-Ab* has high motility compared to P30 ng/mL and control group are correspondingly supported by the finding of Gagliardo et al. [42]. Figure 9 shows the in vivo study of phenotypic effect and relative mRNA expression of M100 ng/mL on the F-AL stage. The simple control group and EtOH (Olive oil-ethanol) show normal pregnancy at 6 dpi (days post-infection) but F-AL of M100 ng/mL treated mice group did not show pregnancy and only see embryos inside of the F-AL body (Figure 9A). M100 ng/mL showed up-regulation of the *Ts-MAPRC2* gene as compared with EtOH (Olive oil-ethanol) and the simple control (*p* ≤ 0.05) (Figure 9B), these findings are amply supported by Gagliardo et al. and Chen et al. [19,42].

## 5. Conclusions

To conclude, this study showed that mifepristone has a profound antagonistic impact on the *Ts-MAPRC2* and has an abortifacient effect on the female adult stage of *T. spiralis.* Moreover, mifepristone treatment downregulated the expression of *Ts-MAPRC2* gene in a dose-dependent manner, which in turn leads to the abortion in pregnant female adult worms in vivo as well as in vitro. To the best of our knowledge, this is the first study regarding cloning, expression of the *rTs-MAPRC2* gene, its expression level, and phenotypic effects with sex hormones (P4 and RU486) at all developmental stages. This would open a new horizon about antihormone drug design, and vaccine therapy of recombinant (*rTs-MAPRC2*) protein with their combined effect to control *Trichinellosis*.

## Data Availability

All data generated or analyzed during this study are included within the article and its additional information file.
